# Small Ruminant Parturition Detection Based on Inertial Sensors—A Review

**DOI:** 10.3390/ani14192885

**Published:** 2024-10-07

**Authors:** Pedro Gonçalves, Maria R. Marques, Shelemia Nyamuryekung’e, Grete H. M. Jorgensen

**Affiliations:** 1 Escola Superior de Tecnologia e Gestão de Águeda and Instituto de Telecomunicações, Universidade de Aveiro, 3810-193 Aveiro, Portugal; 2 Instituto Nacional de Investigação Agrária e Veterinária I.P. (INIAV), Avenida Professor Vaz Portugal, 2005-424 Vale de Santarém, Portugal; rosario.marques@iniav.pt; 3 Division of Food Production and Society, Norwegian Institute of Bioeconomy Research (NIBIO), PB 115, N-1431 Ås, Norway; shelemia.nyamuryekunge@nibio.no (S.N.); grete.jorgensen@nibio.no (G.H.M.J.)

**Keywords:** automatic parturition detection, small ruminants, accelerometer

## Abstract

**Simple Summary:**

Animal births, much like human births, can encounter complications that threaten the well-being of both the mother and the offspring. While monitoring the birth process is essential for ensuring proper care, human supervision can be costly. Commercial systems do exist for large animals, but there are currently no comparable solutions for small ruminants, despite various research efforts. This study explores the application of inertial sensors to detect parturition in small ruminants. This study also highlights which factors most significantly influence the outcomes of each investigation and summarizes the main results regarding birth detection. The review shows that different approaches focus on recognizing particular animal behaviors and designing detection algorithms. Although none of the studies presented a completely reliable detection method, most produced encouraging results, indicating noticeable behavioral changes in the hours preceding birth.

**Abstract:**

The birth process in animals, much like in humans, can encounter complications that pose significant risks to both offspring and mothers. Monitoring these events can provide essential nursing support, but human monitoring is expensive. Although there are commercial monitoring systems for large ruminants, there are no effective solutions for small ruminants, despite various attempts documented in the literature. Inertial sensors are very convenient given their low cost, low impact on animal life, and their flexibility for monitoring animal behavior. This study offers a systematic review of the literature on detecting parturition in small ruminants using inertial sensors. The review analyzed the specifics of published research, including data management and monitoring processes, behaviors indicative of parturition, processing techniques, detection algorithms, and the main results achieved in each study. The results indicated that some methods for detecting birth concentrate on classifying unique animal behaviors, employing diverse processing techniques, and developing detection algorithms. Furthermore, this study emphasized that employing techniques that include analyzing animal activity peaks, specifically recurrent lying down and getting up occurrences, could result in improved detection precision. Although none of the studies provided a completely valid detection algorithm, most results were promising, showing significant behavioral changes in the hours preceding delivery.

## 1. Introduction

Animal birth is a highly significant event for the animals’ welfare [1] and for the sustainability of livestock farming activities [2]. Like in humans, the process can suffer complications that can have severe repercussions for both the offspring and their mothers. Dystocia [3] is a prevalent factor contributing to prolonged parturitions, which traditionally leads to increased risk of neonatal mortality/injuries. In the mothers, the consequences of dystocia or prolonged parturition are many, for example, vaginal prolapses [4] or, in extreme cases, death. Monitoring the process enables the implementation of a parturition nursing practice, therefore avoiding the aforementioned issues. However, the lack of reliable predictions means that a costly surveillance process is still required.

Considerable effort, both from academia [5,6,7,8,9,10] and industry [11,12,13] sectors, has been invested in the past decade to develop an automatic parturition detection method. There are already some commercial products [11,12,13] that perform automatic detection, but they have only been designed for cattle, probably due to the higher economic value of these animals. For small ruminants, several attempts at parturition detection have been documented. The proposed solutions were based on monitoring location and proximity between pairs [14,15] and the use of images and inertial sensors [16]. For instance, Paganoni et al. [15] reported a method that relies on measuring the distance between the ewes and the rams to predict the day of parturition. Their calculation accurately predicted the birth date within five days for 100% of the ewes. Over the last decade, inertial sensors have been employed to monitor various aspects of animal life due to their affordability, minimal intrusiveness with animal life, and their ability to easily identify behavior patterns [17]. They have been used in different scenarios, such as monitoring behavior [18,19], activity [20], well-being [21], and parturition events [4,22].

The present paper aims to evaluate the application of wearable inertial sensors for the detection and prediction of small ruminant parturition. Thus, the objective of this systematic literature review is to identify the key methodologies, the most promising techniques used, and the main findings documented in the literature. This review addresses the main scientific questions as follows:

RQ 1: How were the animals monitored in terms of the employed devices and their application?

RQ 2: What was the duration, extent of animal handling, and level of supervision of the monitoring essays?

RQ 3: Which parturition indicators were studied in those studies?

RQ 4: Which data analysis techniques and features did the authors use?

Including the introduction (Section 1), this review is organized into five sections. Section 2 describes the materials and methods, while Section 3 presents the results. The paper continues with the discussion of the findings (Section 4), and Section 5 concludes the paper by presenting some future perspectives in the field of parturition detection research.

## 2. Materials and Methods

**Figure 1:**
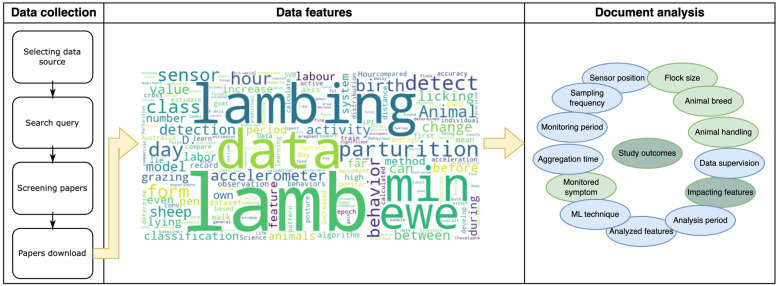
Illustration of the methodology used for data collection, keywords searched as data features, and the document analysis.

The present study comprises a systematic literature review carried out on various information sources, including Pubmed, ScienceDirect, Web of Science, Scopus, and Google Scholar. The methodology is illustrated in Figure 1.

In the study, keywords related to the kidding or lambing process and their synonyms were used for goats and sheep using inertial sensors through the research query ((sheep OR goat) AND (lambing OR calving OR kidding OR parturition) AND detection AND (accelerometer OR gyro OR imo OR mems)).

Table 1 summarizes the paper count results per information source, such as a distribution of papers by species as well as the distribution of papers by a set of events related to animal life. As the initial results returned duplicate entries, and they included papers related to cattle, sheep, and goats, but after a paper screening process, just papers related to the original criteria were kept.

Table 2 identifies the papers retrieved from each of the information sources, divided between goats and sheep.

The analytical procedure continued by examining the text of the “Material and Methods” section, wherein the terms present in the text were identified, and the frequency of occurrence of each word was recorded. The word cloud at the center of Figure 1 graphically illustrates the results obtained. Finally, in the third step, the compilation of words was examined and categorized, generating a list of data features present in the paper data, as depicted on the right side of Figure 1.

## 3. Results

This section presents the findings of the analysis of the obtained documents. It considers the key aspects of the parturition process, encompassing the pattern and changes in typical animal behaviors over the days preceding delivery, the monitoring methodology, and, finally, how the data were analyzed.

### 3.1. Parturition Process

Parturition encompasses a series of chronological events, as described in Table 3.

According to Arnold et al. [34], animals tend to reduce their activity levels in the days prior to giving birth, displaying longer periods of rest. This behavior can be easily monitored using inertial sensors. Each of the articles in this review examined one or more aspects of animal activity. For instance, Smith et al. [27] focused on analyzing the activity patterns throughout the entire parturition process.

The parturition process in both goats and sheep begins with the animal’s seeking isolation from their peers to find a safe place with space to give birth. Accurate detection of this behavior depends on the monitoring method used, such as tracking individual locations or assessing proximity communication between devices worn by each individual. Within the reviewed documents, only one of the studies sought to detect this behavior [26].

Another common behavior involves an increase in the number of lying and standing bouts, usually occurring 12 h prior to parturition. This behavior is due to the discomfort felt by the animals. While most studies refer to this behavior, none of them specifically analyze the changes between the lying and standing bouts. Instead, they analyze the time spent within each category separately [3,16,22].

The final stage of the process is signaled by the dam experiencing noticeable abdominal contractions, often accompanied by the dam’s tendency to lift her head during this period. Although various studies designate this behavior as a common occurrence before the moment of expulsion, only Gurule et al. [3] identified its occurrence based on video records and interpreted it as an indication of labor. Expulsion is the final stage of the parturition process and, according to the literature, lasts for 30–45 min [3]. Typically, it is a gradual process characterized by interspersed contractions, aiding the delivery of the offspring. However, this process would not be so easily detectable by inertial sensors; hence, none of the analyzed work paid attention to it. After the expulsion of viable offspring, and sometimes after a brief moment of rest, the mothers begin to lick their offspring, a process that often lasts up to two hours. Considering the prolonged duration and distinct pattern of the licking behavior, it can be used as an indicator of the completion of the expulsion process. This phenomenon has been analyzed in several studies [3,22,33] and Table 3 synthesizes the events in their chronological order.

The change in animals’ typical behavior patterns, such as those summarized in Table 4 and also referred to in the literature [34], is commonly associated with the dam undergoing some stage of the parturition process. Some studies have employed behavior classification strategies based on accelerometer data and have used the classified behavior information to detect signs of birthing [33].

Smith et al. [27]’s approach was to identify lambing time through the analysis of datasets based on animal activity. In contrast, Fogarty et al. [16] created an ethogram composed of five states (Grazing, Standing, Walking, Lying, and Active behavior) to correlate with the lambing time. Additionally, Sohi et al. [33] and Gurule et al. [3] used ethograms to classify animal behavior. In the three works [3,16,33], each behavior category was aggregated to an accumulative time spent by the animal spanning over the hours around the time of birth. A standard deviation of the time spent in each behavior category across all observed animals around the time of birth was also analyzed. The authors concluded that the differences between peers would invalidate the use of cumulative behavioral time changes as a means of identifying birth. Table 4 summarizes behaviors monitored around parturition time.

### 3.2. Monitoring Essay

The studies involving animal monitoring primarily focused on Merino sheep. Gurule et al. [3] use the Debouillet breed, while Sohi et al. [33] monitored Merino, Border Leicester, and East Friesian breeds. Two studies examined local goat breeds: Kim et al. [23] monitored Korean Native Black Goats, and Gonçalves et al. [25] monitored Charnequeira goats. The studies covered a wide range of animal numbers, with Gurule et al. [3] monitoring 13 animals, while Sohi et al. [33] monitored 165 sheep. The duration of the monitoring periods was up to 3 weeks. However, Gonçalves et al. [25] did not monitor all animals during the same period due to the process of reusing collars on other animals, resulting in a monitoring duration of 33 days.

The handling of the animals during the tests in most of the cases took place inside the barn/shelter to facilitate the supervision of births. Night vision video cameras were implemented for most of the supervision. In the case of Gonçalves et al. [25], direct visual observation was carried out, enabling data acquisition on some of the animal’s parturition in the paddock.

Telemetry information was primarily collected using accelerometer sensors, except for Gonçalves et al. [25], where an IMO was utilized. A few studies reported the use of store-on-board loggers, from which the data were extracted after the deployment period. In contrast, other studies used sensors with communication support, such as Bluetooth Low Energy [22,33] or other forms of radio communication [35], for real-time or near-real-time data acquisition.

The positioning of the sensors in the reviewed studies was on various locations of the animal body. Fogarty et al. [16,26] and Gurule et al. [3] placed the sensor on the ear tag [33], while Turner et al. [22] mounted the sensors on the halter. Smith et al. [27] used a collar to place the sensor on the left side of the neck, and Gonçalves et al. [25] used a collar to support the sensor at the bottom side of the animal’s neck. Whereas Kim et al. [23] mounted the sensor on the goat’s legs.

In terms of operational frequency, accelerometers/IMO were operated at very different frequencies. Gonçalves et al. [25] monitored accelerations at 0.1 Hz, Smith et al. [27] at 10 Hz, Fogarty et al. [16] and Gurule et al. [3] at 12.5 Hz, and Sohi et al. [33] and Turner et al. [22] operated the accelerometer at 30 Hz. Kim et al. [23] reported non-monotonic sampling since their accelerometer operated at 0.1 Hz when no activity was detected and increased in frequency to 1 Hz when activity was detected.

### 3.3. ML Approach

In terms of features analyzed, the reviewed studies followed three main strategies: (i) identifying the typical changes in behavior patterns indicating parturition in animals and looking for a correlation between these behaviors and the time of birth; (ii) extracting activity-related metrics from raw sensor data and searching for correlation; and (iii) using additional information obtained from other sources such as location, meteorological, or ultrasound sensor information. Table 5 represents an extensive list of features used by the studies under analysis for the detection of parturition.

Throughout the reviewed studies, in terms of data analysis duration, varying period lengths were analyzed. Gonçalves et al. [25] reported the shortest period length of analysis, which consisted of the interval between the 5 h before and the hour after birth. Meanwhile, Smith et al. [27] reported the analysis length of the entire trial period, which began 17 days before birth. In addition, different intervals were also used for data aggregation (epoch): some studies tested various data aggregation intervals [16,22,23,26], and aggregation intervals were reported to be between 5 s [33] and 1 min [25].

In algorithmic terms, the choices were predominantly made on Machine learning algorithms, using algorithms based on Concept Drift, Random Forest, Decision Trees, Classification and Regression Tree, Support Vector Machine, and some deep learning algorithms such as CNN, KNN, Naïve Bayes, and XGBoost. Conversely, Smith et al. [27] focused on using several convolutional methods to identify time of birth.

The most effective characteristics in the identification of birth are closely linked to the monitoring strategy adopted (Table 6). For instance, in studies where the monitoring strategy focused on the changes in behavior patterns throughout the birth process, researchers found that changes in behavior throughout the time of birth and the evolution of the occurrence of walking behavior were identified as strong indicators of the birth event [16]. They also identified the behavior of licking the offspring as an indicator of the completion of parturition. Additionally, in studies that analyzed measures related to accelerations [3,22,23,25], variations in accelerations related to the ewe’s discomfort were identified as prominent features. However, according to the location data analyzed by Fogarty et al. [26], extraction of the distance values between individuals in the herd was the most important feature in the recognition process.

In terms of identifying the birthing event, the reviewed studies reported varying results. Some works presented obvious signs of the occurrence of the event without trying to predict the event. Some of the works detected the day of the event with some precision but were unable to identify the specific time [16,25,33]. Among the studies that attempted to identify the time of the event, Smith et al. [27] successfully identified the time of the event within a margin of less than 4 h of error. Kim et al. [23] reported a detection rate of 82.4% of parturitions, with a prediction time of 90.6 ± 99.12 min before the birth time. Additionally, Turner et al. [22] achieved 84.8% accuracy in isolating labor and licking, with 78.1% accuracy in predicting licking behavior.

## 4. Discussion

Automatic parturition detection is a line of work with enormous promise in terms of animal welfare and reduced workload for the farmer. This analysis of the state of the art allowed us to identify a set of studies with very promising results but which do not yet allow to detect the event. An automatic parturition detector, to be useful on a livestock farm, needs to predict the event with acceptable accuracy and sometime in advance, preferably allowing the nursing staff to travel to the location. Even so, the work demonstrated the validity of automatic detection based on the sensing of inertial values.

The different applications of sensors on animals did not show variations in the results obtained, indicating that future solutions could place the sensor in the most convenient location, not necessarily on the ear. This flexibility could enable the sensors to increase in size and weight, extending their energy autonomy and allowing integration into devices that perform multiple monitoring functions across various scenarios.

The studies reviewed were conducted within a limited set of geographical areas, which naturally impacted the available breeds. A prospective automatic mechanism must account for the diverse characteristics of different breeds, such as size and average activity. To generalize the findings and to obtain an efficient detector in different conditions and animal breeds, monitoring trials should be repeated in various locations and with different breeds. The handling of the animals also varied across studies, with some conducted in the paddock and others in the barn. The analysis does not indicate how the place of birth influences animal behavior or the learning process needed to develop a parturition detection mechanism. Regardless, establishing a learning process—whether through statistical methods or machine learning—requires precisely annotated accelerometry data, making video imagery essential. Collected video images can monitor accelerometry data and verify data supervision by a second person, helping to correct any inaccuracies. Naturally, video recording is much easier when animal handling takes place in the barn.

The analysis of the effect of the different sampling frequencies of the sensors made it possible to verify the existing differences but did not conclusively show how the frequency affected the accuracy of the learning model. This is because different identification strategies may require different frequencies as well as different aggregation periods (epochs). Eventually, the establishment of the minimum frequency and data aggregation interval will depend on experience, and these values can only be determined after a successful process of developing an identification algorithm. Additionally, the variations in monitoring and data analysis length periods should not affect the quality of the results, as the monitoring periods in all cases are significantly longer than the durations when signs of parturition are evident.

The strategy for detecting parturition based on behavior is the most important factor for the successful development of a detection mechanism, as it influences all subsequent decisions. Choosing a strategy similar to Turner et al. [22], which is based on identifying behaviors, requires a careful selection of states to be included in the ethogram to ensure accuracy, as behaviors can have dynamic similarity. The correlation between the frequency of behaviors over the hours before birth varies among animals, as indicated in several studies [16,33]. On the other hand, the use of metrics derived from raw acceleration data also depends on differences between animals [3], such as age and weight, and may require a prior standardization process of the data.

Probably the most promising monitoring strategy may consist of monitoring the effects of discomfort in the moments before birth and frequent standing/laying bouts. This approach assumes analyzing maximum and minimum acceleration measurements or their deviations within aggregation intervals. Implementing this strategy could also help to identify periods of reduced activity before labor and potentially detect the behavior of licking the offspring, which is common postpartum and notably observed in Turner et al.’s study [22].

Differences in activity between pairs may depend on the age, weight, and possibly the breed and are very visible in different studies. The pre-processing of data must include a data standardization mechanism to mitigate this effect; otherwise, the classification model learning process will be seriously impacted. Additionally, a functional detection mechanism must be tested with animals of different species and eventually updated with monitoring data from these species; otherwise, the classification algorithm will not be generalizable.

Finally, the quality of the data used has a huge impact on the quality of the learning model, including the supervision data. The use of video recordings has a huge impact on this quality as it allows careful annotation of accelerometry data, as well as checking the quality of this annotation whenever necessary.

## 5. Conclusions

Automatic detection of small ruminant parturition is of utmost importance for animal welfare and for improving the profitability of livestock farms, but there is still no system capable of triggering nursing help when needed. This paper presents a systematic review of the state of the art, aiming to analyze the various reported attempts of automatic detection of small ruminant parturition using inertial sensors.

The results obtained allowed us to understand that the sensors used consisted mainly of accelerometers operating at frequencies between 0.1 and 30 Hz, although the sensors were placed in various locations on the animals’ bodies. The trials lasted between 4 days and 3 weeks, and some of the animals were monitored in the barn or in the shelter, given the greater ease of using video images for continuous data monitoring.

Monitoring strategies differed across studies, with some studies focusing on classifying behavior and the evolution of behaviors throughout the lambing or kidding period, others on defining and monitoring a metric of animal welfare throughout the period, and most studies analyzing metrics associated with animal activity throughout the period.

Data analysis techniques varied between studies and included techniques based on statistical analysis, Machine learning techniques, and even deep learning. In terms of the most impactful features, most studies identified metrics based on the variation of acceleration data associated with animal movement and the discomfort inherent in the birthing process.

## Figures and Tables

**Table 1:** Number of papers obtained from the reference databases Pubmed, ScienceDirect, Web of Science, Scopus, and Google Scholar by research topic and species.

Number of Papers Retrieved	Pubmed	ScienceDirect	Web of Science	Scopus	Google Scholar *
Total	23	24	16	11	105
Activity/Behavior	1	0	2		18 (11)
Feeding	1	1	1		7 (4)
Grazing	2	1	1	1	13 (8)
Health and welfare	1	1	1		4 (3)
Kidding	1	0	2	1	(3)
Lambing	3	4	5	6	(11)
Lameness	1	1	1		(2)
Parasites	3	2	2		(2)
Reproduction	0	0	0	1	6 (5)
Reviews					
Livestock					21
Small ruminants	1	0	0		(16)
Other species	2	11	0		26
Calving		4			10
Non-relate papers	7	3	1	2	0
Small ruminant **	13	10	15	11	49
Kidding or lambing	4	4	7	7	14

* 1320 papers since 2015. Papers were sorted by relevance, and the first six pages were considered and clean from duplicates; in brackets is the number of papers regarding small ruminants. ** Without reviews.

**Table 2:** The distribution of papers relevant to small ruminants across the different information sources used.

	Pubmed	ScienceDirect	Web of Science	Scopus	Google Scholar	Total of Unique Papers
Kidding	[23]		[23,24]	dataset	[23,24,25]	3
Lambing	[3,16,26]	[3,16,22,27]	[3,16,22,26]		[3,16,22,26,28,29,30,31,32,33]	11

**Table 3:** Parturition-related events chronology in ewes.

Chronological Order	Event/Behavior	Detection	Duration	Reference
1	Lower activity	Measurement through acceleration values	Days before	[3,26,27]
2	Isolation from the flock	Difference between peer localizations	12 h	[3,26]
3	Lying and standing bouts	Accelerometer values pattern	12 h	[3,16]
4	Contractions	Accelerometer values pattern	30–45 m	[3]
5	Expulsion		30–45 m	[3]
6	Licking newborn	Posture classified by accelerometer information	Up to 2 h after expulsion	[3,22,33]

**Table 4:** The changes in behavior patterns around parturition in sheep.

Behavior	Beginning–End	Homogeneity between Animals	Reference
Grazing	Drop 12 h before lambing recovery at 12 post-lambing	Changes do not occur at the same hours for all animals	[16,33]
Ruminating	Decline in rumination from 8 h to 4 h post-lambing	Changes do not occur at the same hours for all animals	[33]
Walking	Increased until the previous day and then returned to the same number of minutes per hour from the following day	Significant peak at the time of lambing	[33]
Idle	Sharp drop at the time, gradual recovery after 4 h post-lambing	Wide variation	[33]
Standing	Increased on the day of lambing	Significant drop on the day	[3]
Laying	Decreased on the day of lambing and kept lower after it	Slight decrease from the day	[16]
Activity	Decrease of activity		[3,16]
Licking	Peaked 4 h after lambing, then fell back sharply	Changes do not occur at the same hours to all animals	[33]

**Table 5:** Dataset features used by the reviewed studies for detection of parturition.

Feature	Associated Symptom	Reference
Behavior evolution	Behavior evolution around lambing time	[33]
Activity/Motion Magnitude Vector	$Act=\sqrt{{Acc}_{x}^{2}+{Acc}_{y}^{2}+{Acc}_{z}^{2}}$	[33]
Raw accelerations	Body dynamics around delivery time	
Mean accelerations	Mean acceleration value of x,y,z axes within each epoch	[3,33]
Average accelerations	Average acceleration value of x,y,z axes within each epoch	[19]
Max accelerations	Maximum acceleration value of x,y,z axes within each epoch	[3]
Min accelerations	Minimum acceleration value of x,y,z axes within each epoch	[3]
Kurtosis	$k_{l}=\frac{\frac{1}{n}\;\sum_{i=1}^{n}{\left(x_{i}-\bar{x}\right)}^{4}}{{\left(\frac{1}{n}\sum_{i=1}^{n}{\left(x_{i}-\bar{x}\right)}^{2}\right)}^{2}}$	[33]
Standard deviation	Standard variation for x, y, z, values within epoch	[33]
Average Standard deviation	${SD}_{ZYX}=\frac{1}{T}\;\sum_{t=1}^{T}\left(SDX+SDY+SDZ\right)$	[19]
Power signal	$y=\frac{norm{\left(X\right)}^{2}}{sample\;rate}$	[33]
Peak-to-peak amplitude	Maximum-to-minimum difference	[33]
Signal Magnitude Area	$\frac{1}{T}(\sum_{t=1}^{T}\left(\left|\;Ax(t)\right|\right)+\sum_{t=1}^{T}\left(\left|Ay(t)\right|\right)+\sum_{t=1}^{T}\left(\left|\;Az(t)\right|\right))$	[3]
Entropy	$\frac{1}{T}(\sum_{t=1}^{T}\left({1+x\left(t\right)+y\left(t\right)+z\left(t\right))\;}^{2}\right){ln(1+x\left(t\right)+y\left(t\right)+z\left(t\right))\;}^{2})$ *	[19]
Energy	$\frac{1}{T}(\sum_{t=1}^{T}\left(({x(t)}^{2}\right)+\left({y(t)}^{2}\right)+\left({z(t)}^{2}\right))$ *	[19]
Movement Intensity	$MI=\frac{1}{T}(\sum_{t=1}^{T}\sqrt{\left(Ax^{2}+Ay^{2}+Az^{2}\right)}(t)$ *	[3]
Movement variation	$\frac{1}{T}(\sum_{t=1}^{T}\left(\left|x_{t-1}-x_{t}\right|\right)+\left(\left|y_{t-1}-y_{t}\right|\right)+\left(\left|z_{t-1}-z_{t}\right|\right))$ *	[19]
Labor Pain Index	Metric that combines the amount of side-lying and leg-extending behavior with the amount of activity	[23]
Weather data	Average air temperature, hourly rainfall, average wind speed, and average solar radiation	[26]
GGNS data	Mean, max, and min speed, mean distance between ewes, closest ewe, and minimum convex polygon	[26]
Time spent active	Time spent active, from behavior analysis	[26]
Pitch, Roll	Pitch, roll angles taken from gyro: $\begin{array}{c} Pitch=arctan\left(\frac{x}{\sqrt{{(y}^{2}+z^{2})}}\right)\\ Roll=arctan\left(\frac{y}{\sqrt{{(x}^{2}+z^{2})}}\right) \end{array}$	[25]
Distance to ground	Ultrasound-measured distance between collar and ground	[25]

* where T is the total number of counts in the epoch.

**Table 6:** Summary of sensor type, monitoring essay, monitoring period, and dataset analysis most relevant characteristics featured in studies aiming to detect parturition in small ruminants.

Reference	Gurule et al. [3]	Forgarty et al. [16]	Turner et al. [22]	Kim et al. [23]	Gonçalves et al. [25]	Fogarty et al. [26]	Smith et al. [27]	Sohi et al. [33]
*Publication date*	April 2021	May 2020	May 2023	Feb 2024	Mar 2024	Jan 2021	Jun 2020	Jul 2022
Sensor type								
*Accelerometer*	Axivity AX3		Actigraph	iBS03, INGICS TECHNOLOGY		Axivity AX3	HAM-x16	ActiGraph wGT3X-BT
*Position*	Ear Tag		Halter mounted	Leg placed	Collar placed	Ear Tag	Collar left placed	Halter mounted
*Frequency*	12.5 Hz		30 Hz	1 Hz when active or 0.1 Hz	0.1 Hz	12.5 Hz	10 Hz	30 Hz
*Data storage*	Logger		Not defined, but with BLE support		Gateway	Logger	Logger	Not defined, but with BLE support
Monitoring essay								
*Animals*	13 ewes	27 ewes	101 ewes	17 goats	16 goats	35 training ewes 33 testing ewes	76 multiparous ewes	32 and 165 ewes
*Breeds*	Debouillet	Merino cross	Merino	Korean Native Black Goats	Charnequeira	Merino-cross	Merino	Merino, Border Leicester and East Friesian
*Origin*	New Mexico, USA	South Island of New Zealand	Australia	Republic of Korea	Portugal	South Island of New Zealand	Australia	
*Monitoring* *period*	7 and 14 days	10 days before to 2 days after	2–3 weeks before parturition	Not defined	33 days, collars used in a rotation basis	7 days prior 6 days post lambing	Up to 17 days before parturition	12 days
*Animal handling*	On a paddock	Barn Paddock and shelter	shelter	Paddock and barn	Paddock and barn	Barn	Paddock	Paddock
*Animal supervision*	Video recordings 2–3 h each day	Night vision cameras	Daylight hours video recordings	RGB cameras	Direct visual observation	Night vision cameras	Night vision cameras	Human observations and video recording
Dataset analysis								
*Analysis period*	30 days	7 days and 12 h before and after lambing	Some before lambing work start, some after lambing	12 h before the parturition	5 h before parturition	Entire period		400 h
*Epochs/Segment size*	10 s, 10 s aggregated to 1min	10 s and 30 s epochs	5, 10, 20, 30, and 60 s epochs	8, 10, 12, and 14 min	1 min epochs	10 s, 30 s epochs	30 segments	5 s epoch
*ML approach*	Activity metrics and an ethogram analysis	Behavior and activity related metrics analysis	Behavior evolution analysis	Calculate LPI	Concept drift for detection of changes, DL model for learning algorithm	Usage GGNS with inertial and weather data	Evolution of MMV over the period	Activity related metrics analysis
*ML technique*	Random forests model	SVM, CART, LDA	LSTM, SVM, RF, CNN	SVM and DT over LPI	Concept drift, DT, SVC, LR, RF, KNN, Naïve Bayes, XGBoost	SVM with LOOCV	max-val, 2-stage, D-conv, disc-conv and log-conv, combine 8 different segment sizes L = 0.5, 1, 2, 3, 4, 6, 8, 12 h were considered	SVM, and a DL/neural network approach
*Analyzed features*	Max, Mean, Min, MI, R, SD SMA of accelerations, accumulated time in a set of behaviors	Daily and hourly variation of the time spent in any of the behaviors	300 features based on a set of behaviors	LPI Mean, Standard deviation of accelerations	Pitch, roll, min, hour, accelerations, distance to ground	Mean, min., Max. speed; Mean distance to peers; Closest peer; Min. convex polygon; Time spent grazing, lying, standing, walking, active; Posture changes; Average air temperature, wind speed, and solar radiation	Motion magnitude vector	Mean, Kurtosis, Standard deviation, Power signal, Peak-to-peak amplitude, and five behavior ethograms
*Impacting* *features*	Metrics directly calculated from the accelerometer	Posture changes around lambing, Hourly walking behavior	Detection of licking behavior as a good indicator for the time of birth	X- and Z-axis acceleration values	Pitch, Roll, Min, Hour	GNSS (MDP.Mean, CP, MDPP) and postural changes	MMD and EMD equally performed	Mean, SD, kurtosis, power signal, peak-to-peak amplitude, autocorrelation, PCA
*Most relevant outcomes*	Metrics calculated directly from the accelerometer such as the range in the X-axis change more impacting that predicted behaviors	Daily and hourly changes in sheep behavior at parturition	84.8% accuracy with isolation of labor and licking, licking prediction with 78.1% accuracy	Detection rate of 82.4% of parturitionsprediction time was 90.6 ± 99.12 min before the birth time	Day detection with 61% accuracy	Detected 84% of the dates. Showed feasibility 12 h before	MAE of 5.33 h 66% of ewes had parturition estimates with errors of 4 h or less. 84% of ewes had parturition estimates with error lower than 12 h	Established relationships between accelerometer-based behaviors/time around lambing as lambing time approached 10 days before lambing, with widths of ~15–20 h.

Legend: BLE—Bluetooth Low Energy; RGB—Red Green Blue; LPI—Low Probability of Intercept; DL—Deep Learning; GGNS—Gradient-Guided Nested Sampling algorithm; MMV—Multiple Measurement Vectors; SVM—Support Vector Machine learning algorithm; CART—Classification Tree; LDA—Linear Discriminant Analysis; LSTM—Long Short-Term Memory; RF—Random Forests; CNN—Convolutional Neural Networks; DT—Decision Trees; SVC—Support Vector Classifier; LR—Logistic Regression; KNN—K-Nearest Neighbors; LOOCV—Leave-One-Out Cross-Validation; MI—Movement Intensity; R—Range; SD—Standard Deviation; SMA—Signal Magnitude Area; GNSS—Global Navigation Satellite System; CP—Closest Peer; MDP— Mean Distance to Peers; MMD—Maximum Mean Discrepancy; EMD—Earth Movers Distance; MAE— Mean Absolute Error; PCA—Principal Component Analysis.

## Data Availability

Data are contained within the article.
